# Programmable synthetic engineering of adaptive microbial cell factories: from amide transforming enzymes to advanced biocatalytic systems

**DOI:** 10.3389/fbioe.2026.1921701

**Published:** 2026-09-29

**Authors:** Syeda Warisul Fatima, Shahenvaz Alam

**Affiliations:** 1 Winship Cancer Institute, School of Medicine, Emory University, Atlanta, GA, United States; 2 Emory Musculoskeletal Institute, School of Medicine, Emory University, Atlanta, GA, United States

**Keywords:** adaptive biofactories, artificial intelligence, guided protein engineering, metabolic engineering, microbial cell factories, microbial enzymes, programmable biocatalysis

## Abstract

Microbial cell factories integrate metabolic engineering, synthetic regulation, protein engineering, and computational design to improve catalytic performance and process robustness. Within this landscape, amide transforming enzymes provide diverse functions ranging from nitrogen metabolism and hydrolysis to covalent protein remodeling. This review examines amidases, asparaginases, glutaminases, nitrilases, nitrile hydratases, acylases, and carbamylases, with particular emphasis on microbial transglutaminase (MTGase). MTGase is distinguished by its ability to catalyze covalent ε-(γ-glutamyl) lysine crosslinking through acyl transfer reactions, enabling protein stabilization, molecular assembly, immobilization, and spatial organization of catalytic systems. This review critically examines the transition from classical amide transforming enzymes toward engineered biocatalytic systems and evaluates how MTGase could be leveraged as a protein level control component in microbial cell factories. Experimental developments in MTGase production, structural and protein engineering, synthetic genetic circuits, CRISPR based regulation, metabolic control, nanobiomaterials, and spatial biocatalysis are evaluated alongside computational approaches including AlphaFold assisted structure prediction, molecular docking, molecular dynamics, machine learning guided mutagenesis, and metabolic modeling. Emphasis is placed on experimentally validated outcomes and current limitations. Key unresolved barriers include intracellular off target crosslinking, proteostatic burden, variable crosslink stoichiometry, limited temporal control of MTGase activity, chassis dependence, and industrial scale up. Where direct evidence is lacking, prospective applications are distinguished from established capabilities and corresponding engineering strategies are proposed. Collectively, this review envisions MTGase as a controllable protein level platform for synthetic biology, while defining the evidence, limitations, and experimental requirements needed for its broader application in microbial biotechnology.

## 1 Introduction

Synthetic biology integrates molecular biology, systems engineering, computational modeling, metabolic engineering, and artificial intelligence (AI) to design microbial systems with defined functions (Poppeliers et al., 2026; Kim et al., 2025). Engineered microbial cell factories support the production of pharmaceuticals, industrial enzymes, fine chemicals, biofuels, therapeutic proteins, biomaterials, and high value metabolites (Jeung et al., 2025; Ren et al., 2025; Liu et al., 2025; Guo et al., 2026). Advances in synthetic genetic circuits, dynamic regulatory systems, CRISPR mediated genome engineering, and metabolic pathway optimization have expanded the capacity of microorganisms for controlled biomanufacturing (Kim et al., 2025; Guo et al., 2026; Qin et al., 2026; Bai et al., 2026).

Microbial cell factories offer advantages over conventional chemical synthesis, including renewable feedstocks, reduced energy requirements, and high catalytic specificity (Cho et al., 2022). However, their industrial performance remains constrained by metabolic burden, synthetic circuit instability, host toxicity, genetic drift, pathway crosstalk, and reduced robustness under changing process conditions (Wehrs et al., 2019; Xie, 2022). These limitations create a need for molecular tools that can couple catalytic function with more precise regulation and metabolic control (Lea Smith et al., 2025).

Amide transforming enzymes have long served as important biocatalysts in industrial and biomedical biotechnology. Amidases, asparaginases, glutaminases, and related enzymes participate in nitrogen metabolism and have applications in pharmaceutical production, food processing, bioremediation, and fine chemical synthesis (Qeshmi et al., 2018; Voor et al., 2025; Rotilio et al., 2025). Most of these enzymes act primarily through hydrolytic amide bond cleavage. Microbial transglutaminase (MTGase), in contrast, catalyzes acyl transfer reactions that generate covalent ε-(γ-glutamyl) lysine bonds between proteins (Beitello et al., 2026; Kieliszek, 2026; Fatima and Khare, 2018). This reaction enables post translational protein modification, stabilization, molecular assembly, and structural organization (Fatima et al., 2019; Fatima et al., 2020; Fatima et al., 2021a), providing functions that differ from those of conventional hydrolytic amide transforming enzymes.

This review therefore examines MTGase beyond its established use as an industrial crosslinking enzyme and considers its potential as a protein level engineering tool in microbial systems. Its catalytic properties have supported applications in protein engineering, biomaterial fabrication, enzyme immobilization, and spatial organization of catalytic systems (Kieliszek, 2026; Fatima et al., 2019; Fatima et al., 2020). Related studies have also explored MTGase based approaches in orthogonal protein modification, extracellular matrix fabrication, and nanobiomaterial systems (Beitello et al., 2026; Fatima et al., 2021a; Mondal et al., 2023). However, the extent to which these capabilities can be transferred to intracellular microbial engineering remains incompletely established and requires careful evaluation of substrate specificity, crosslink stoichiometry, temporal control, host compatibility, and proteostatic burden.

Computational biology and artificial intelligence provide additional tools for addressing some of these engineering constraints (Siddique et al., 2025). Approaches including AlphaFold based structure prediction, machine learning assisted mutagenesis, molecular modeling, and predictive metabolic analysis can support the identification and prioritization of enzyme variants and pathway interventions (Lv et al., 2025; Yang J. et al., 2024). Their relevance to MTGase, however, depends on experimental validation of predicted improvements in activity, specificity, stability, and process performance. Accordingly, this review distinguishes established experimental capabilities from emerging computational strategies and prospective applications, with the aim of defining the requirements for extending MTGase toward controlled use in microbial biotechnology.

### 1.1 Scope of the review

This review examines the functional evolution of amide transforming enzymes from conventional biocatalysts toward engineered components of microbial production systems. The review focuses on microbial transglutaminase (MTGase), whose covalent protein crosslinking activity distinguishes it from classical hydrolytic enzymes and supports protein assembly, immobilization, post translational modification, and spatial organization of catalytic systems.

The review evaluates experimental developments in MTGase production, structural and protein engineering, synthetic genetic circuits, CRISPR based regulation, metabolic control, nanobiomaterials, and spatial biocatalysis. Computational approaches, including AlphaFold assisted structural prediction, machine learning guided engineering, and metabolic modeling, are discussed in relation to experimentally validated outcomes. The review also critically examines unresolved limitations, including substrate specificity, intracellular off target crosslinking, proteostatic burden, crosslink stoichiometry, temporal control of enzyme activity, chassis dependence, and industrial scalability, while distinguishing established capabilities from prospective applications.

Thus, the central thesis of this review is that MTGase may provide a protein level engineering interface between genetic regulation and cellular phenotype. Realization of this potential, however, requires experimentally demonstrated control of substrate specificity, temporal activity, spatial localization, crosslink stoichiometry, and host compatibility. This framework provides the basis for critically distinguishing established MTGase capabilities from emerging and prospective applications in microbial biotechnology.

## 2 Amide transforming enzymes and evolution toward programmable biocatalysis

The amide transforming enzymes have served as foundational biocatalysts in industrial biotechnology and pharmaceutical applications (Lubberink et al., 2023; Marti et al., 2024). Although they are more functionally specialized than newer programmable enzyme platforms, they remain highly valuable because of their catalytic efficiency, substrate selectivity, and established roles in both applied microbiology and therapeutic biochemistry. Recent advances in protein engineering, and systems level metabolic design have substantially improved the performance of these enzymes through strategies such as rational mutagenesis, directed evolution, heterologous expression, codon optimization, and pathway balancing (Shi et al., 2025).

### 2.1 Amidases in microbial biotechnology

Amidases are an important class of hydrolase enzymes that catalyze the cleavage of amide bonds into corresponding carboxylic acids and ammonia (Bayer et al., 2025; Ganjoo and Babu, 2025). These ubiquitous enzymes are distributed across bacteria, fungi, plants, and mammals and play critical roles in nitrogen metabolism, xenobiotic degradation, and microbial adaptation to environmental stress. In industrial biotechnology, microbial amidases have garnered considerable attention due to their catalytic specificity, mild reaction conditions, and environmentally sustainable biotransformation capabilities (Singh R. et al., 2025). Recent advances in microbial metabolic engineering have further expanded amidase applications in green chemistry, pharmaceutical synthesis, and industrial biocatalysis.

Microbial amidases participate in nitrile and amide metabolism, where they catalyze hydrolysis reactions that can contribute to both contaminant degradation and preparative biotransformation (Li et al., 2025). Experimental studies provide direct evidence for these applications. For example, a novel amidase, PsaA, from *Bosea* sp. P5 was shown to initiate degradation of the herbicide propanil through amide bond hydrolysis, and the corresponding strain was incorporated into an artificial microbial consortium capable of further propanil mineralization. More recently, the bifunctional amidase PamC from *Rhodococcus* sp. C1 was shown to completely degrade propanil across concentrations ranging from 10 μg/L to 150 mg/L within 0.33–10 h, while also hydrolyzing several aryloxyphenoxypropionate herbicides. These studies provide direct mechanistic evidence that selected amidases can contribute to pesticide transformation and environmental bioremediation rather than relying solely on inferred enzyme function (Zhang et al., 2023a; Zhou et al., 2024).

Amidases have also demonstrated preparative value in fine chemical synthesis. An amidase signature family enzyme from *Pantoea* sp. was used for hydrolysis of two chloronicotinamide to two chloronicotinic acid, an intermediate used in agrochemical and pharmaceutical synthesis. Under fed batch conditions, the process produced up to 370 mM two chloronicotinic acid with 94.2% substrate conversion, providing quantitative evidence for the synthetic utility of amidase catalysis (Zheng et al., 2018). However, these capabilities are strongly enzyme and substrate dependent, and factors such as substrate specificity, product inhibition, catalytic stability, and process conditions continue to determine practical performance. Thus, amidases should be considered substrate specific biocatalysts with demonstrated utility in selected environmental and synthetic applications rather than universally applicable catalysts.

Recent developments in enzyme engineering have improved amidase catalytic performance through protein engineering, heterologous expression, and pathway optimization strategies (Bayer et al., 2025; Ganjoo and Babu, 2025; Singh R. et al., 2025). Most naturally occurring amidases perform hydrolytic chemistry only, and they are not inherently suited for dynamic structural remodeling or post translational control. Ongoing research, therefore, focuses not only on improving their catalytic efficiency and operational stability, but also on expanding their substrate range, enhancing solvent and temperature tolerance, and embedding them more effectively into synthetic metabolic networks for industrial fine chemical production. Although they contribute significantly to industrial biocatalysis, their utility within programmable synthetic biological systems remains relatively constrained compared with emerging multifunctional enzymatic platforms.

### 2.2 Asparaginases and nitrogen metabolic engineering

Asparaginases represent a specialized subclass of amide transforming enzymes that catalyze the hydrolysis of L-asparagine into L-aspartate and ammonia (Jana et al., 2025; Alam et al., 2018). These enzymes have become particularly important in pharmaceutical biotechnology due to their clinical use in acute lymphoblastic leukemia therapy, where depletion of extracellular asparagine selectively targets malignant lymphoblasts with impaired asparagine biosynthesis capabilities (Avramis and Tiwari, 2006). Microbial L-asparaginases derived from *Escherichia coli* and *Erwinia chrysanthemi* remain among the most widely used therapeutic enzymes in oncology (Chand et al., 2020; Ghasemian et al., 2019).

Beyond therapeutic applications, asparaginases are widely utilized in food biotechnology to reduce acrylamide formation during thermal food processing (Jana et al., 2025). Acrylamide, a potentially carcinogenic compound formed through Maillard reactions between reducing sugars and asparagine, can be significantly reduced through enzymatic pre-treatment using microbial asparaginases. Therefore, these enzymes have gained substantial industrial importance in food safety and sustainable food processing technologies (Mudaliar et al., 2025).

In microbial metabolism, asparaginases also contribute to nitrogen regulation and amino acid homeostasis. Their substrate specificity and catalytic efficiency have made them attractive candidates for metabolic engineering applications aimed at improving nitrogen utilization and biosynthetic pathway optimization (Fisher and Wray Jr, 2002). Nevertheless, similar to amidases, most asparaginase systems remain fundamentally hydrolytic and relatively limited in their ability to support programmable structural remodeling, adaptive catalytic organization, or post translational engineering. These limitations have stimulated widespread interest in multifunctional enzymatic systems capable of extending beyond hydrolysis toward programmable molecular engineering. A major challenge in the application of asparaginases is that naturally occurring enzymes often show undesirable properties, including immunogenicity, insufficient thermal stability, glutaminase associated side activity, or a narrow substrate performance window under industrial conditions (Sonowal et al., 2024; Brumano et al., 2019; Lanvers Kaminsky, 2017). To address these limitations, recent studies have employed semi rational engineering, structure guided mutagenesis, molecular docking, and machine learning assisted design to improve catalytic behavior while reducing unwanted effects (Khan and Khan, 2025). For instance, engineered variants of asparaginase from *Bacillus licheniformis*, including the quintuple mutant ILRAC, have shown marked increases in specific activity and improved thermal stability relative to the wild type enzyme (Zhou et al., 2022). Such studies illustrate how integrated computational and experimental strategies can generate clinically and industrially superior enzymes.

### 2.3 Evolution toward transglutaminase systems

The inclusion of amidase and asparaginase in adaptive microbial cell factories provides a critical comparative framework for understanding the emergence of more multifunctional enzyme platforms such as MTGase (Fatima and Khare, 2018; Khan, 2025). Traditionally, amidases and asparaginases have been regarded as highly specialized catalysts with relatively narrow biochemical functions. However, this view is gradually changing as synthetic biology leverages them into nutrient responsive circuits, metabolic control modules, and chassis engineering strategies. Modern engineering approaches, including directed evolution, PEGylation, active site redesign, and host pathway optimization, extend the utility of these enzymes beyond simple substrate hydrolysis (Zhou et al., 2025; Pagar et al., 2021; Yun et al., 2026). They are now being incorporated into modular genetic systems, automated strain construction workflows, and rapid prototyping pipelines for pathway development. When combined with AI guided protein engineering, CRISPR based transcriptional regulation, and multi-omics analysis, amidase and asparaginase based systems can contribute to the design of more robust, scalable, and responsive industrial bioprocesses.

The integration of traditional amide transforming enzymes with microbial transglutaminase represents a key transition toward programmable biocatalytic systems in synthetic biology. By coupling AI assisted protein design with hierarchical metabolic engineering, researchers are beginning to construct adaptive microbial cell factories capable not only of efficient chemical production but also of controlled post translational protein remodeling. The emergence of microbial transglutaminase has significantly expanded the conceptual and functional landscape of amide transforming enzymes (Doti et al., 2020). Unlike amidases and asparaginases that primarily catalyze hydrolytic reactions, versatile MTGase catalyzes acyl transfer reactions between γ-carboxamide groups of glutamine residues and primary amines, particularly ε-amino groups of lysine residues. This catalytic mechanism results in the formation of stable ε- (γ-glutamyl) lysine covalent crosslinks and the release of ammonia (Fatima and Khare, 2018).

The ability of MTGase to mediate covalent protein crosslinking confers functional capabilities absent in classical hydrolytic enzymes, including programmable biomolecular assembly, protein stabilization, structural remodeling, and adaptive network formation (Fatima et al., 2020; Fatima et al., 2021a). These properties have enabled broad industrial applications in food protein stabilization, biomaterials engineering, tissue scaffolding, enzyme immobilization, and therapeutic protein modification. More recently, researchers have begun exploring MTGase as a programmable molecular tool for orthogonal protein modification, dynamic biomaterial engineering, and adaptive metabolic organization as shown in Figure 1. Figure 1 framework comprehends the functional landscape of the principal amide transforming enzyme classes and connects catalytic chemistry with progressively broader biological and translational functions. Amidases, asparaginases, glutaminases, nitrilases and nitrile hydratases, and acylases and carbamylases are first distinguished according to their characteristic substrates and catalytic transformations. These reactions underpin specific biological functions, including amide hydrolysis, nitrogen regulation, glutamine utilization, nitrile conversion, and acyl compound processing, which subsequently influence broader metabolic processes such as nitrogen metabolism, carbon nitrogen balance, xenobiotic metabolism, and nitrogen assimilation. The framework further illustrates how these catalytic properties can be leveraged in environmental detoxification, fermentation and food processing, metabolic reprogramming, green chemistry, and metabolite detoxification. These functions extend to applications including bioremediation, wastewater treatment, amino acid and fine chemical production, pharmaceutical intermediate synthesis, and precision biocatalysis, together with biomedical applications such as enzyme based leukemia therapy, diagnostic biosensors, modulation of cancer metabolism, prodrug development, and enzymatic therapeutics. Collectively, the framework establishes a reaction to metabolism to application continuum that provides the biochemical foundation for the subsequent cell factory engineering framework.

**FIGURE 1:**
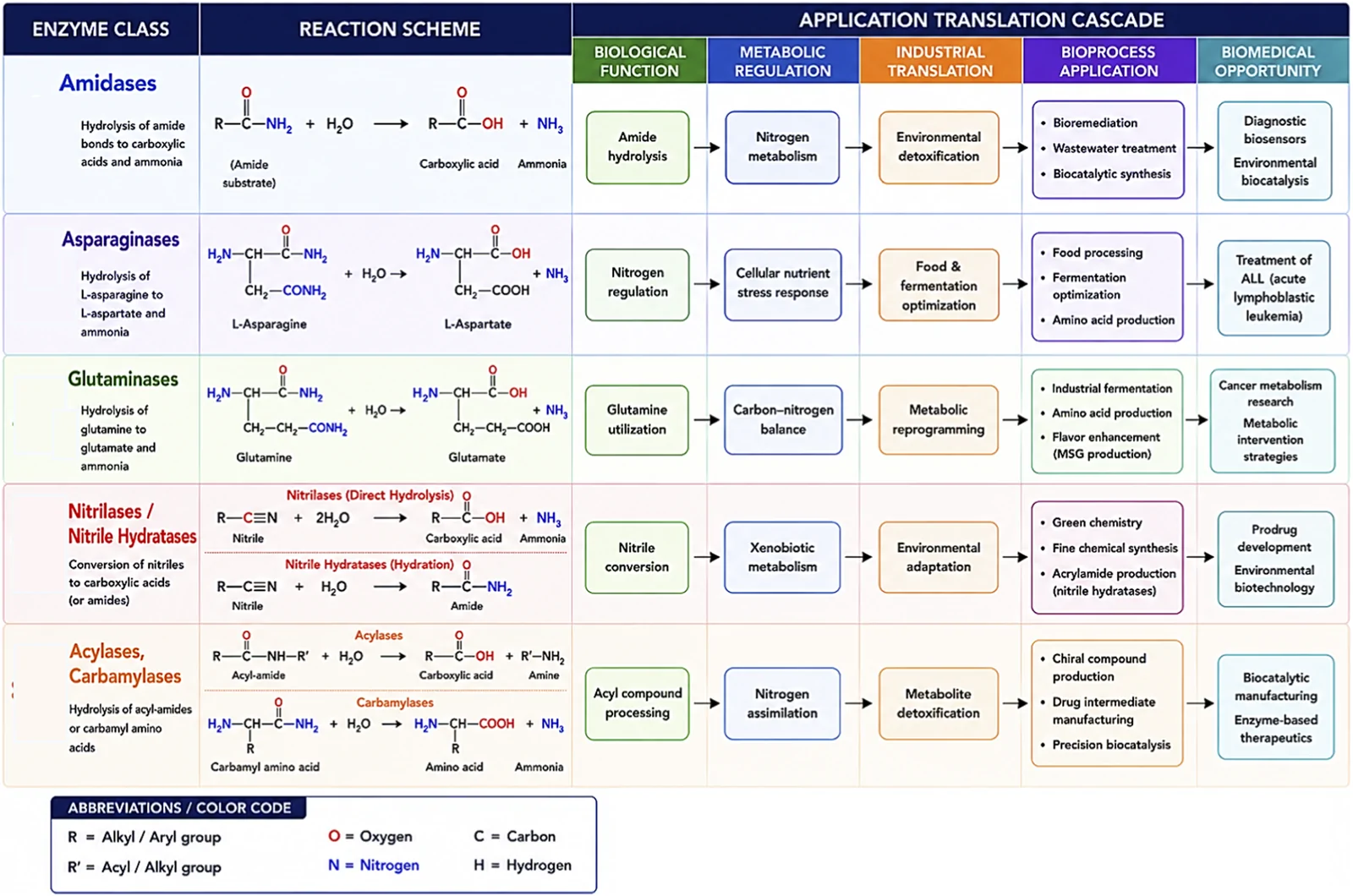
Functional landscape of amide transforming enzymes: Reaction chemistry, metabolic functions, and translation into industrial and biomedical applications. Comparative overview of the major classes of amide transforming enzymes, including amidases, asparaginases, glutaminases, nitrilases/nitrile hydratases, and acylases/carbamylases. The figure summarizes their representative catalytic reactions, primary biological functions, metabolic regulatory roles, industrial applications, bioprocess implementations, and emerging biomedical opportunities, illustrating how enzyme chemistry is translated into programmable biocatalytic functions across synthetic biology, industrial biotechnology, environmental engineering, and therapeutic development.

Importantly, MTGase mediated crosslinking represents a transition from conventional catalytic hydrolysis toward programmable post translational engineering. By enabling covalent remodeling of proteins and biomolecular assemblies, MTGase supports the development of synthetic catalytic architectures capable of dynamic structural organization and adaptive functionality. These emerging applications position MTGase as a next-generation programmable enzymatic platform with substantial potential in synthetic biology and microbial engineering (Zhou et al., 2025).

### 2.4 Other emerging programmable biocatalysts in synthetic biology and adaptive microbial engineering

Beyond classical amide transforming enzymes and MTGase, several microbial biocatalysts contribute complementary functions to synthetic biology and microbial cell factory engineering (Doti et al., 2020). These enzyme classes are considered here primarily as comparative benchmarks for the different levels of control available in synthetic biology, ranging from metabolic conversion to protein assembly and genome regulation. Together, nitrilases, nitrile hydratases, acylases, carbamylases, glutaminases, proteases, sortases, glycosyltransferases, ligases, recombinases, and CRISPR associated nucleases illustrate how catalytic, structural, and regulatory functions can be combined within engineered biological systems (Table 1).

**TABLE 1:** Comparative functional, industrial, biomedical, and synthetic biology characteristics of amide transforming enzymes and emerging programmable biocatalytic platforms.

Enzyme class	Catalytic activity/Core reaction	Representative microbial sources	Natural biological function	Representative industrial applications	Biomedical and therapeutic applications	Synthetic biology utility	Structural and functional advantages	Major limitations	Engineering strategies	References
Amidases	Hydrolysis of amide bonds into carboxylic acids and ammonia	*Pseudomonas*, *Rhodococcus*, *Bacillus*, *Alcaligenes*	Nitrogen metabolism, xenobiotic degradation, environmental detoxification	Bioremediation, wastewater treatment, nitrile conversion, pharmaceutical intermediate synthesis, fine chemical production	Limited direct therapeutic use; primarily industrial biocatalysis	Useful for metabolic pathway engineering, biodegradation circuits, and biosensing applications	High catalytic specificity, environmentally friendly catalysis, broad substrate range, mild reaction conditions	Limited structural programmability, poor suitability for adaptive regulation, primarily hydrolytic function	Directed evolution, active site engineering, heterologous expression, pathway optimization	(Rotilio et al., 2025; Bayer et al., 2025; Ganjoo and Babu, 2025; Singh et al., 2025a; Li et al., 2025; Zhang et al., 2023a; Zhou et al., 2024; Zheng et al., 2018)
Asparaginases	Conversion of L- asparagine into L-aspartate and ammonia	*Escherichia coli*, *Erwinia chrysanthemi*, *Bacillus* spp.	Nitrogen regulation and amino acid metabolism	Food processing to reduce acrylamide formation, fermentation optimization	Widely used for treatment of acute lymphoblastic leukemia and emerging cancer therapies	Applicable in nutrient responsive synthetic circuits and metabolic regulation systems	Clinically validated enzyme platform, strong substrate specificity, established industrial production systems	Immunogenicity, narrow substrate specificity, limited structural engineering capability	PEGylation, recombinant engineering, mutagenesis, protein stabilization	(Jana et al., 2025; Alam et al., 2018; Avramis and Tiwari, 2006; Chand et al., 2020; Ghasemian et al., 2019; Mudaliar et al., 2025; Fisher and Wray Jr, 2002; Sonowal et al., 2024; Brumano et al., 2019; Lanvers Kaminsky, 2017; Khan and Khan, 2025; Zhou et al., 2022)
Glutaminases	Hydrolysis of glutamine into glutamate and ammonia	*Bacillus*, *Aspergillus*, *Corynebacterium*	Nitrogen homeostasis, amino acid metabolism, carbon nitrogen balance	Flavor enhancement, glutamate production, fermentation biotechnology	Cancer metabolism studies, metabolic intervention strategies, therapeutic research	Supports metabolic rewiring and adaptive nutrient utilization pathways	Central role in cellular metabolism, compatible with industrial fermentation systems	Primarily metabolic function with limited structural engineering potential	Metabolic engineering, pathway balancing, recombinant overexpression	(Ryu et al., 2026; Gibhardt et al., 2026)
Nitrilases	Direct conversion of nitriles into carboxylic acids and ammonia	*Rhodococcus*, *Pseudomonas*, *Arthrobacter*	Xenobiotic metabolism and detoxification	Green chemistry, pharmaceutical synthesis, agrochemical production	Limited therapeutic relevance	Useful in engineered biodegradation pathways and sustainable manufacturing	High catalytic efficiency, environmentally sustainable processing	Limited multifunctionality and structural programmability	Directed evolution, substrate redesign, active site optimization	(Xia et al., 2024; Menon et al., 2025; Jiang et al., 2026)
Nitrile Hydratases	Hydration of nitriles into corresponding amides	*Rhodococcus*, *Pseudomonas*, *Brevibacterium*	Nitrile utilization and environmental adaptation	Large scale acrylamide production and industrial nitrile biotransformations	Minimal biomedical applications	Useful in synthetic biotransformation pathways	Exceptional industrial scalability and catalytic turnover	Limited versatility outside nitrile chemistry	Catalytic redesign and stability engineering	(Menon et al., 2025; Jiang et al., 2026)
Acylases	Hydrolysis of acyl amide bonds and acyl group transfer reactions	*Bacillus*, *Pseudomonas*	Amino acid metabolism and cellular detoxification	Antibiotic production, chiral chemical synthesis, pharmaceutical manufacturing	Drug intermediate production	Moderate utility in engineered metabolic pathways	High catalytic selectivity and process robustness	Limited adaptability and narrow substrate range	Protein engineering and directed evolution	(Bravo et al., 2025)
Carbamylases	Hydrolysis of carbamyl amino acids into amino acid products	*Agrobacterium*, *Pseudomonas*	Nitrogen metabolism and amino acid processing	Production of optically pure amino acids and specialty chemicals	Limited biomedical applications	Useful in precision biosynthetic pathways	High enantioselectivity and industrial relevance	Narrow substrate spectrum	Rational design and active site optimization	(Martínez Rodríguez et al., 2010)
Proteases	Hydrolysis of peptide bonds within proteins and peptides	Diverse bacterial and fungal species	Protein turnover, nutrient acquisition, stress adaptation	Food processing, detergents, peptide production, industrial biotechnology	Therapeutic enzymes, wound management, digestive supplements	Frequently used in synthetic biology for regulated protein degradation and circuit control	Exceptional catalytic diversity and industrial robustness	Risk of non specific protein degradation	Stability engineering and substrate specificity optimization	(Alsahari et al., 2025)
Sortases	Site specific protein ligation and covalent protein conjugation	*Staphylococcus* spp. and related bacteria	Cell wall protein anchoring	Protein conjugation, enzyme immobilization, vaccine production	Therapeutic protein engineering and vaccine development	Powerful tool for orthogonal protein assembly and molecular engineering	Highly specific covalent protein ligation and modular assembly capability	Restricted substrate recognition motifs	Rational redesign and substrate engineering	(Amacher and Antos, 2024)
Glycosyltransferases	Transfer of carbohydrate moieties onto proteins, lipids, and metabolites	Various bacteria, fungi, and actinomycetes	Glycan biosynthesis and cell envelope construction	Glycoengineering, biopharmaceutical manufacturing, specialty chemical production	Glycoprotein therapeutics and vaccine development	Advanced pathway engineering and molecular diversification	Enables structural complexity and product diversification	Complex substrate requirements and pathway interactions	AI guided glycoengineering and pathway optimization	(Liang et al., 2015)
Ligases	Covalent joining of biomolecules and macromolecular assembly	Diverse microbial systems	DNA repair, biosynthesis, cellular assembly processes	Synthetic pathway construction, molecular assembly technologies	Biomolecule conjugation and therapeutic engineering	Supports construction of synthetic biological modules and circuits	Efficient and programmable covalent assembly	Often energy dependent and context sensitive	Rational engineering and directed evolution	(Ritsema et al., 2025)
Recombinases	DNA rearrangement, integration, excision, and recombination	Phage derived and bacterial systems	Genome maintenance and genetic adaptation	Synthetic genetic circuits and genome engineering	Gene therapy and genome editing research	Central tools for programmable DNA level control	Precise and programmable genome manipulation	Potential off target recombination events	Recombinase redesign and orthogonal system development	—
CRISPR Nucleases	Programmable DNA and RNA recognition, cleavage, and regulation	Bacterial adaptive immune systems	Genome defense and antiviral protection	Genome engineering, strain development, precision biotechnology	Gene therapy, precision medicine, diagnostic platforms	Foundational platform for modern synthetic biology	Exceptional programmability and sequence specificity	Delivery limitations and off target concerns	Guide RNA engineering, protein redesign, AI assisted optimization	(Sarkar et al., 2023; Wu et al., 2020; Dinh and Prather, 2019)
Microbial Transglutaminase (MTGase)	Acyl transfer reactions between glutamine and lysine residues generating covalent protein crosslinks	*Streptomyces mobaraensis*, *Streptomyces hygroscopicus* and recombinant hosts	Protein stabilization, structural remodeling, extracellular matrix organization	Food biotechnology, enzyme immobilization, biomaterials, tissue scaffolds, catalytic materials	Drug delivery systems, regenerative medicine, therapeutic biomaterials	Enables programmable post translational engineering, protein circuit design, spatial metabolic organization, and adaptive biomolecular assembly	Covalent programmable protein remodeling, broad substrate compatibility, calcium independent activity, robust structural stabilization, modular engineering potential	Metabolic burden in heterologous hosts and limited systems level optimization studies	Rational design, directed evolution, synthetic circuit integration, thermostability engineering, AI guided redesign	(Beitello et al., 2026; Kieliszek, 2026; Fatima and Khare, 2018; Fatima et al., 2019; Fatima et al., 2021a; Mondal et al., 2023; Zhou et al., 2025; Pagar et al., 2021; Yun et al., 2026; Doti et al., 2020; Chan et al., 2023; Tan et al., 2026; Akbari et al., 2021; Shi et al., 2026; Wang et al., 2025; Zheng et al., 2025; Song et al., 2026; Deweid et al., 2019; Cui et al., 2025; Hu et al., 2025; Savoca et al., 2018; Conibear, 2020; Zhu and Wu, 2024; Duarte et al., 2020)
Engineered MTGase Platforms	Programmable covalent biomolecular assembly, adaptive catalytic architecture formation, orthogonal protein engineering	Synthetic microbial chassis and engineered biological systems	Artificial catalytic network formation and adaptive biomolecular organization	Precision fermentation, smart biomaterials, engineered living materials, programmable catalytic systems	Advanced therapeutic biomaterials, adaptive drug delivery systems, regenerative bioengineering	Central platform for post translational synthetic biology, orthogonal protein programming, intelligent biofactory construction, and adaptive cellular engineering	Adaptive catalytic interfaces, programmable molecular organization, structural modularity, compatibility with AI guided optimization and systems engineering	Requires advanced systems integration, regulatory control, and biosafety optimization	CRISPR integration, AI guided mutagenesis, digital twin modeling, dynamic feedback circuits, synthetic promoter engineering	(Beitello et al., 2026; Yun et al., 2026; Chan et al., 2023; Tan et al., 2026; Wang et al., 2025; Zheng et al., 2025; Song et al., 2026; Deweid et al., 2019; Cui et al., 2025; Hu et al., 2025; Ma et al., 2019; Gajšek et al., 2019; Wang et al., 2019; Zhang et al., 2023c; Trengove et al., 2021; Okawa et al., 2022; Chang et al., 2025; Aqeel et al., 2024; Fatima et al., 2021b)

Nitrilases and nitrile hydratases are established industrial biocatalysts for nitrile conversion and sustainable chemical manufacturing (Xia et al., 2024). Nitrilases directly convert nitriles into carboxylic acids, whereas nitrile hydratases generate the corresponding amides, supporting applications in pharmaceutical synthesis, agrochemical production, environmental remediation, and green chemistry (Menon et al., 2025; Jiang et al., 2026). Acylases and carbamylases similarly provide selective transformations relevant to pharmaceutical intermediates, antibiotics, specialty chemicals, and optically pure amino acids (Bravo et al., 2025; Martínez Rodríguez et al., 2010). Glutaminases occupy a different metabolic role by regulating glutamine conversion, nitrogen homeostasis, and carbon nitrogen balance, thereby influencing nutrient utilization and metabolic flux in microbial systems (Ryu et al., 2026). Collectively, these enzymes mainly provide catalytic and metabolic functions that can be incorporated into engineered pathways, but their capacity for direct structural or regulatory control is comparatively limited.

At the protein level, proteases, sortases, glycosyltransferases, and ligases provide more direct control over protein abundance, modification, and molecular assembly. Engineered proteases can regulate protein levels through controlled degradation and can therefore support synthetic circuits requiring rapid protein level responses (Alsahari et al., 2025). Sortases enable site specific protein ligation and have been applied in protein engineering, enzyme immobilization, vaccine development, and biomaterial construction (Amacher and Antos, 2024). Glycosyltransferases expand molecular diversity through transfer of carbohydrate moieties to proteins, lipids, and metabolites, supporting glycoengineering of therapeutic and specialty biomolecules (Liang et al., 2015). Ligases similarly enable covalent biomolecular assembly and modular protein or peptide engineering (Ritsema et al., 2025). These systems provide useful comparators for MTGase because they demonstrate how post translational enzymatic reactions can extend cellular engineering beyond metabolic conversion alone.

At the genomic and regulatory level, recombinases and CRISPR associated nucleases provide programmable control of DNA organization and gene expression (Sarkar et al., 2023; Wu et al., 2020). Recombinases support DNA integration, excision, inversion, and rearrangement, whereas CRISPR based technologies enable targeted genome editing and transcriptional regulation. Their integration with metabolic engineering provides upstream control over pathway composition and gene expression, but these systems primarily operate at the genetic or transcriptional level rather than directly reorganizing protein assemblies.

Overall, these enzyme classes represent complementary levels of biological engineering rather than independent technological themes. Nitrilases, nitrile hydratases, acylases, carbamylases, and glutaminases primarily support catalytic and metabolic control; proteases, sortases, glycosyltransferases, and ligases extend engineering to protein processing and molecular assembly; and recombinases and CRISPR systems provide genetic and regulatory control. Within this hierarchy, MTGase occupies a distinct position because its acyl transfer activity enables covalent post translational protein remodeling and biomolecular assembly. This comparison provides the conceptual basis for examining how protein level engineering may complement metabolic and genetic regulation in microbial cell factories (Khan and Khan, 2025).

### 2.5 Conceptual shift toward programmable biocatalytic systems

The progression from amidases and asparaginases toward transglutaminase systems reflects a broader transition in biotechnology from primarily hydrolytic catalysis toward more integrated forms of molecular engineering. Traditional biocatalysts generally function as catalytic components within defined biochemical pathways, whereas emerging synthetic biological systems increasingly combine catalytic activity with structural organization and regulatory control (Capecchi et al., 2026).

This transition has contributed to the development of post translational synthetic biology, which extends biological engineering beyond transcriptional and translational regulation to direct modification of proteins and biomolecular assemblies. Within this framework, MTGase mediated covalent crosslinking provides a means of constructing protein networks, catalytic interfaces, extracellular architectures, and biomaterial systems through controlled protein modification (Maddock et al., 2020).

Systems level biocatalysis further emphasizes spatial metabolic organization, enzyme compartmentalization, substrate channeling, and dynamic pathway regulation. MTGase based approaches may contribute to these objectives through covalent biomolecular assembly and structural stabilization, although their direct application to intracellular metabolic organization remains to be established experimentally. Collectively, the progression of amide transforming enzymes toward more integrated biocatalytic systems reflects the convergence of enzymology, synthetic biology, systems biotechnology, biomaterials science, and computational engineering (Table 2).

**TABLE 2:** Evolution from classical amide transforming enzymes to programmable biocatalytic systems: Functional transition toward adaptive microbial cell factories.

Evolutionary stage	Dominant enzyme classes	Primary biological function	Engineering capability	Synthetic biology integration	Representative technologies	Major limitations	Next evolutionary step	References
Classical Biocatalysis	Amidases, Asparaginases, Glutaminases	Hydrolysis of amide containing substrates	Catalytic conversion	Minimal	Bioremediation, food processing, amino acid metabolism	Limited programmability and structural control	Multifunctional enzyme engineering	(Bayer et al., 2025; Ganjoo and Babu, 2025; Singh et al., 2025a; Li et al., 2025; Zhang et al., 2023a; Zhou et al., 2024; Zheng et al., 2018; Jana et al., 2025; Alam et al., 2018; Avramis and Tiwari, 2006; Chand et al., 2020; Ghasemian et al., 2019; Mudaliar et al., 2025; Fisher and Wray Jr, 2002; Sonowal et al., 2024; Brumano et al., 2019; Lanvers Kaminsky, 2017; Khan and Khan, 2025; Zhou et al., 2022; Ryu et al., 2026)
Industrial Biotransformation	Nitrilases, Nitrile Hydratases, Acylases, Carbamylases	High efficiency chemical conversion	Process optimization	Low	Green chemistry, pharmaceutical synthesis, specialty chemicals	Limited adaptive regulation	Dynamic metabolic engineering	(Xia et al., 2024; Menon et al., 2025; Jiang et al., 2026; Bravo et al., 2025; Martínez Rodríguez et al., 2010)
Protein Processing and Modification	Proteases, Peptidases, Lipases, Esterases	Protein and biomolecule modification	Modular catalytic functions	Moderate	Protein processing, immobilization, industrial biotechnology	Poor pathway coordination	Spatial enzyme organization	(Alsahari et al., 2025)
Molecular Assembly and Protein Engineering	Sortases, Ligases, Glycosyltransferases	Covalent assembly and molecular diversification	Site specific biomolecular engineering	High	Protein conjugation, glycoengineering, biomaterials	Limited systems level integration	Programmable protein networks	(Amacher and Antos, 2024; Liang et al., 2015; Ritsema et al., 2025; Hu et al., 2025; Wang et al., 2019; Zhang et al., 2023c; Trengove et al., 2021; Okawa et al., 2022)
Genetic and Regulatory Engineering	Recombinases, DNA Polymerases, CRISPR associated nucleases	Genome modification and regulatory control	Programmable genetic manipulation	Very High	Synthetic circuits, genome engineering, CRISPR regulation	Primarily transcriptional control	Post translational engineering	(Sarkar et al., 2023; Wu et al., 2020; Yang et al., 2024b; Ream and Prather, 2024; Dinh and Prather, 2019)
Programmable Post Translational Engineering	Microbial Transglutaminase (MTGase)	Covalent protein remodeling and adaptive molecular assembly	Dynamic protein level control	Very High	Protein scaffolds, biomaterials, enzyme clustering, spatial biocatalysis	Limited orthogonal control systems	Intelligent adaptive biofactories	(Beitello et al., 2026; Fatima and Khare, 2018; Zhou et al., 2025; Pagar et al., 2021; Yun et al., 2026; Doti et al., 2020; Chan et al., 2023; Tan et al., 2026; Akbari et al., 2021; Shi et al., 2026; Wang et al., 2025; Zheng et al., 2025; Song et al., 2026; Deweid et al., 2019; Cui et al., 2025; Hu et al., 2025; Savoca et al., 2018; Conibear, 2020; Zhu and Wu, 2024; Duarte et al., 2020)
Adaptive Biocatalytic Systems	Engineered MTGase platforms integrated with CRISPR, AI, and metabolic engineering	Autonomous biomolecular organization and adaptive regulation	Multi layer programmable control	Extremely High	Precision fermentation, synthetic organelles, engineered living materials	Systems integration challenges	Digital twin biofactories	(Qin et al., 2026; Zheng et al., 2025; Song et al., 2026; Singh et al., 2025b; Chang et al., 2025; Aqeel et al., 2024; Fatima et al., 2021b)
Intelligent Microbial Cell Factories	AI guided programmable catalytic ecosystems	Self optimizing biological manufacturing	Autonomous design build test learn cycles	Transformative	Generative AI, predictive metabolic modeling, autonomous biomanufacturing	Regulatory and biosafety challenges	Next-generation adaptive synthetic biology	(Qin et al., 2026; Singh et al., 2025b; Shukla et al., 2026; Cochrane et al., 2026; Douchi et al., 2026; Chen et al., 2025; Groff Vindman et al., 2025; Jun et al., 2025; Mariam et al., 2025)

## 3 Structural biology and catalytic mechanism

### 3.1 Catalytic mechanism of MTGase

Microbial transglutaminase catalyzes acyl transfer reactions between the γ carboxamide groups of glutamine residues and primary amine donors, most commonly lysine residues within proteins. The reaction results in the formation of stable ε-(γ-glutamyl) lysine isopeptide bonds accompanied by ammonia release (Fatima and Khare, 2018). This catalytic mechanism enables irreversible covalent crosslinking between proteins, contributing to enhanced structural stability, resistance to proteolytic degradation, and formation of higher order biomolecular networks.

The catalytic cycle typically begins with nucleophilic attack on the glutamine side chain by the catalytic cysteine residue within the enzyme active site, generating a thioester intermediate. Subsequently, lysine amino groups or alternative primary amine donors attack the intermediate to form covalent crosslinks. In the absence of suitable amine acceptors, water molecules may act as nucleophiles, resulting in deamidation reactions.

Thus, this catalytic versatility enables MTGase to support diverse applications in biomaterials engineering, protein stabilization, enzyme immobilization, and synthetic biological systems (Fatima et al., 2020; Fatima et al., 2021a; Mondal et al., 2023). Importantly, covalent crosslinking mediated by MTGase provides opportunities for programmable protein assembly and adaptive structural organization, distinguishing MTGase from conventional hydrolytic amide transforming enzymes.

### 3.2 Structural features and active site architecture

Most industrially relevant MTGase enzymes are derived from *Streptomyces* species, particularly *Streptomyces mobaraensis* (Fatima and Khare, 2018; Fatima et al., 2019). Unlike mammalian transglutaminases, microbial transglutaminases are calcium independent, smaller in molecular size, and structurally more suitable for industrial engineering applications (Kieliszek, 2026). The catalytic core typically contains conserved active site residues essential for acyl transfer activity and substrate recognition.

Structural analyses have demonstrated that MTGase possesses a compact globular architecture with accessible catalytic pockets that facilitate interaction with diverse protein substrates. The enzyme exhibits relatively broad substrate specificity, enabling crosslinking of various proteins, peptides, and biomaterials. Conformational flexibility and accessible catalytic surfaces contribute to its utility in industrial biotechnology and synthetic biology applications (Kieliszek, 2026; Fatima and Khare, 2018).

Advancements in structural biology have provided deeper mechanistic insights into enzyme structure, catalytic activity, substrate specificity, and regulatory interactions. High resolution crystallography, molecular dynamics simulations, and computational docking studies have enabled rational design strategies aimed at improving catalytic efficiency, thermostability, and substrate specificity.

### 3.3 Protein engineering approaches

Protein engineering strategies have substantially expanded the industrial and synthetic biology applications of MTGase (Zhou et al., 2025). Directed evolution approaches involving iterative mutagenesis and high throughput screening have generated enzyme variants with improved thermal stability, catalytic efficiency, solvent tolerance, and pH adaptability. Rational mutagenesis guided by structural modeling has further enabled optimization of active site accessibility and substrate selectivity (Chan et al., 2023; Tan et al., 2026). Representative experimental milestones in MTGase production, immobilization, protein engineering, and emerging biotechnological applications are summarized in Table 3.

**TABLE 3:** Representative experimental milestones in the engineering and emerging applications of microbial transglutaminase.

Research area	Study focus	Synthetic biology tool/Approach	Application	Major advancement	Future potential	References
Enzyme Production	MTGase production using agro industrial waste	Fermentation optimization	Industrial enzyme production	Demonstrated low cost substrate utilization for MTGase production	Process optimization and scale up	(Fatima et al., 2019)
Enzyme Immobilization	MTGase immobilization on porous magnetic nanoparticles	Magnetic nanoparticle immobilization	Reusable biocatalysis	Improved catalyst recovery and retained activity after repeated use	Reusable MTGase biocatalysts	(Ma et al., 2019)
Enzyme Immobilization	Transglutaminase immobilization on modified magnetic nanoparticles	Carrier based immobilization	Cleaner production technologies	Improved apparent activity and operational stability	Reusable catalytic systems	(Gajšek et al., 2019)
Bioconjugation	Enterokinase immobilization mediated by MTGase	Site specific covalent conjugation	Immobilized enzyme systems	Retained high enterokinase activity after immobilization and reuse	Site specific enzyme assembly	(Wang et al., 2019)
Nanobiotechnology	MTGase immobilization on multiwalled carbon nanotubes	Nanomaterial assisted immobilization	Hydrogel scaffold fabrication	Combined enzyme immobilization with crosslinked scaffold formation	Biomaterial and catalytic scaffold development	(Fatima et al., 2020)
Nanomedicine	MTGase nanoflowers for breast cancer theranostics	Enzyme inorganic hybrid nanostructures	Cancer theranostics	Demonstrated MTGase nanoflower based biomedical application	Application specific nanomedicine platforms	(Fatima et al., 2021a)
Expression Regulation	Transcriptional regulation of MTGase expression in *Streptomyces* mobaraensis	Regulator engineering	MTGase production	Identified regulatory interventions that augment MTGase transcription	Improved host level production control	(Fatima et al., 2021b)
Biomaterials	MTGase reinforced gelatin methacryloyl hydrogels	Enzymatic crosslinking with photocrosslinking	Cartilage tissue engineering	Improved *ex vivo* adhesion and hydrogel stability	Hybrid crosslinking biomaterials	(Trengove et al., 2021)
Biomaterials	Hyaluronan and gelatin hydrogel crosslinked using an MTGase reactive Link module	Sequence directed enzymatic crosslinking	Extracellular matrix inspired hydrogels	Enabled covalent network formation with retained biological function	Sequence controlled biomaterial assembly	(Okawa et al., 2022)
Molecular Delivery	Transglutaminase polyethyleneimine nanoflowers for anti miR 210 delivery	Nanoflower mediated molecular delivery	Glioblastoma therapy research	Demonstrated cellular delivery of anti miR 210	Targeted molecular delivery systems	(Mondal et al., 2023)
Protein Engineering	Semi rational mutagenesis of microbial transglutaminase	Structure guided mutagenesis	Catalytic optimization	Improved MTGase substrate activity	More selective and efficient MTGase variants	(Chan et al., 2023)
Bioconjugation	PNGase F immobilization mediated by MTGase	Site specific covalent immobilization	Reusable glycoenzyme systems	Preserved enzymatic performance with improved reuse and thermal stability	Controlled enzyme immobilization	(Zhang et al., 2023c)
Heterologous Production	Efficient MTGase production in Komagataella phaffii	Expression and secretion engineering	Recombinant enzyme production	Integrated strategies improved heterologous MTGase production	Host optimized manufacturing	(Aqeel et al., 2024)
Systems Biotechnology	Multi omics analysis of high MTGase production and co culture fermentation	Multi omics and fermentation engineering	MTGase biomanufacturing	Linked production phenotype with multi omics mechanisms and co culture strategy	Data informed process optimization	(Chang et al., 2025)
Enzyme Immobilization	Site directed enzyme immobilization using transglutaminase catalyzed bioconjugation	Site directed immobilization	Immobilized biocatalysts	Demonstrated controlled immobilization on microporous resins	More reproducible enzyme orientation and reuse	(Hu et al., 2025)
Machine Learning	iCASE guided optimization of MTGase	Structure informed machine learning	Industrial enzyme engineering	R215L MTGase reached 109.99 U/mg specific activity	Data driven mutation prioritization	(Zheng et al., 2025)
Substrate Specificity	Engineering MTGase substrate preference for nanobody labeling	Docking, virtual mutagenesis, and molecular dynamics	Site specific protein labeling	Y278E shifted substrate preference and enabled single site nanobody labeling	Orthogonal protein modification	(Wang et al., 2025)
Bioconjugation	Site specific and oriented antibody immobilization using MTGase	Enzymatic antibody conjugation	Biosensor and surface functionalization	Expanded MTGase scope for oriented immobilization of native antibodies	Controlled biomolecular interfaces	(Beitello et al., 2026)
Thermostability Engineering	Helix corner motif engineering of *Streptomyces* mobaraensis MTGase	Folding free energy guided protein engineering	Industrial biocatalysis	Improved MTGase thermal stability through structure guided engineering	More robust process enzymes	(Tan et al., 2026)
Artificial Intelligence	AI guided thermostability engineering of MTGase	Artificial intelligence and molecular dynamics	Enzyme optimization	S199E increased half life and catalytic activity	AI assisted MTGase engineering	(Song et al., 2026)
Orthogonal Bioconjugation	Dual payload conjugation using endoglycosidase and MTGase	Orthogonal enzymatic conjugation	Antibody drug conjugates	Demonstrated efficient dual payload antibody conjugation	Modular orthogonal bioconjugation	(Yun et al., 2026)

Thermostability engineering remains particularly important for industrial bioprocessing applications requiring prolonged catalytic activity under elevated temperatures. Similarly, pH optimization strategies have facilitated improved compatibility with diverse fermentation systems and biomaterial fabrication processes. Engineering efforts targeting substrate specificity may also enable orthogonal and programmable protein modification systems for synthetic biology applications (Fatima and Khare, 2018).

Engineering biology approaches have facilitated the heterologous expression of MTGase in diverse microbial chassis, including *E. coli, Bacillus subtilis*, and yeast systems, enabling scalable biomanufacturing and seamless integration into synthetic metabolic pathways (Akbari et al., 2021).

### 3.4 AI guided structural biology and computational engineering

The rapid maturation of computational protein engineering has expanded the available strategies for identifying structural determinants, prioritizing mutations, and predicting enzyme function. Structure prediction platforms such as AlphaFold2 and AlphaFold3 provide high quality models that can support identification of active site geometry, substrate binding regions, conformational constraints, and candidate mutation sites (Shi et al., 2026). However, structure prediction alone does not establish catalytic improvement. Molecular docking, virtual mutagenesis, molecular dynamics, and machine learning are therefore increasingly used as complementary approaches to connect structural information with experimentally measurable properties such as activity, substrate specificity, and thermostability.

Recent MTGase studies provide direct evidence for this progression. (Wang et al., 2025) used molecular docking, Rosetta based virtual mutagenesis, and molecular dynamics to redesign the substrate preference of a thermostable *S. mobaraensis* transglutaminase variant. The Y278E substitution shifted the activity ratio for the nonpreferred GGGGQR substrate relative to CBZ Gln Gly from 0.05 to 0.93 and enabled single site labeling of GGGGQR tagged nanobodies, although the computational screening success rate was approximately 10%, highlighting the continued requirement for experimental validation (Wang et al., 2025). More recently, Zheng et al. (2025) developed the machine learning based iCASE strategy to optimize industrial enzymes and validated its applicability to MTGase. The R215L MTGase variant reached a specific activity of 109.99 U/mg, exceeding a previously reported combinatorial variant and demonstrating that structure informed machine learning can identify productive single residue substitutions (Zheng et al., 2025). Song et al. (2026) used artificial intelligence guided thermostability engineering further identified E164L, E164P, S199E, and S199Q as beneficial MTGase variants. Among these, S199E exhibited 2.3 fold and 5.8 fold longer half-lives at 60 °C and 50 °C, respectively, together with a 1.4 fold increase in enzyme activity. Molecular dynamics analysis was subsequently used to investigate the structural basis of the improved stability (Song et al., 2026).

These studies demonstrate that computational engineering of MTGase has progressed beyond structural prediction toward experimentally validated optimization of stability and substrate recognition. Nevertheless, AlphaFold, docking, molecular dynamics, and machine learning address different stages of the design process and should not be considered equivalent. AlphaFold primarily provides structural hypotheses, docking estimates enzyme substrate interactions, molecular dynamics examines conformational behavior, and machine learning can prioritize mutations based on learned sequence or structure property relationships. Generative protein design remains less extensively validated for MTGase and should therefore be considered an emerging strategy rather than an established engineering approach.

## 4 MTGase as a programmable synthetic biology tool

Microbial transglutaminase has emerged as a versatile molecular platform capable of extending synthetic biology beyond conventional transcriptional engineering toward programmable post translational control and adaptive biomolecular organization (Deweid et al., 2019). Unlike classical hydrolytic enzymes that function primarily through substrate conversion, MTGase enables covalent remodeling of proteins and biomolecular assemblies through acyl transfer mediated crosslinking reactions (Fatima and Khare, 2018). This catalytic versatility allows MTGase to function not only as a biocatalyst but also as a programmable molecular engineering tool supporting structural stabilization, adaptive biomaterial formation, metabolic organization, and orthogonal protein modification. These properties support investigation of MTGase as a protein level engineering tool that may complement transcriptional and metabolic regulation in microbial systems (Beitello et al., 2026).

### 4.1 Programmable protein engineering and molecular assembly

Among the most distinctive attributes of MTGase is its capacity to drive programmable protein engineering and modular molecular assembly, thereby expanding the functional design space of engineered biological systems. Through covalent ε- (γ-glutamyl) lysine bond formation, MTGase enables stable and site-specific protein crosslinking that can be utilized for constructing engineered protein networks, catalytic scaffolds, immobilized enzyme systems, and tunable biomaterials (Beitello et al., 2026; Fatima and Khare, 2018). These properties provide important advantages over conventional non covalent protein assembly strategies that are often susceptible to environmental instability and proteolytic degradation (Shi et al., 2026).

Orthogonal protein engineering is actively directed toward the development of programmable post translational modification systems capable of selectively regulating biomolecular interactions without perturbing endogenous cellular pathways (Cui et al., 2025). In this context, MTGase mediated covalent crosslinking provides a robust mechanism for engineering orthogonal protein architectures with enhanced structural stability and catalytic precision. Synthetic protein networks generated through MTGase mediated assembly may facilitate spatial organization of metabolic pathways, enzyme clustering, and adaptive catalytic interfaces within microbial hosts.

MTGase also plays a vital role in enzyme immobilization and structural stabilization strategies (Fatima et al., 2020). Immobilized enzyme systems have attracted growing attention in industrial biotechnology due to their improved catalytic durability, substrate accessibility, and reusability under large scale bioprocessing conditions. MTGase assisted immobilization may improve enzyme retention and catalytic efficiency within biomaterial matrices while reducing proteolytic degradation and pathway instability (Beitello et al., 2026; Hu et al., 2025). Such approaches may support the development of modular biocatalytic platforms for precision fermentation and sustainable industrial manufacturing.

Additionally, MTGase enabled protein assembly systems are being investigated for fabrication of tunable biomaterials and adaptive catalytic matrices (Zhou et al., 2025). These biomaterials may possess customizable mechanical properties, substrate responsiveness, and programmable structural organization suitable for applications in biosensing, tissue engineering, therapeutic delivery, and microbial bioprocessing. The integration of MTGase with programmable biomolecular systems therefore represents an important step toward adaptive and multifunctional synthetic biological platforms (Savoca et al., 2018).

### 4.2 Post translational synthetic biology

Synthetic biology has historically focused on transcriptional and translational regulation through promoter engineering, CRISPR mediated genome editing, and RNA based control systems. However, increasing metabolic complexity and dynamic cellular environments have highlighted the limitations of exclusively transcription centered engineering strategies (Deweid et al., 2019). Post translational synthetic biology is emerging as a complementary paradigm aimed at regulating biological systems directly at the protein and biomolecular assembly level (Conibear, 2020).

Within this framework, MTGase provides a potential enzymatic mechanism for covalent post translational protein modification and assembly (Deweid et al., 2019). By catalyzing covalent protein remodeling and crosslinking reactions, MTGase enables covalent structural organization and stabilization of protein assemblies beyond conventional gene expression control mechanisms. Such capabilities may facilitate adaptive protein circuitry, programmable catalytic networks, and orthogonal molecular systems capable of responding to metabolic or environmental perturbations (Zhu and Wu, 2024).

Enzymatic remodeling systems mediated by MTGase may also support dynamic cellular organization through spatial regulation of catalytic interfaces and metabolic modules (Duarte et al., 2020). Protein level engineering may provide several advantages over traditional transcriptional control, including faster response kinetics, improved structural stability, reduced pathway interference, and direct regulation of enzymatic functionality. Consequently, MTGase mediated post translational systems may contribute to the development of adaptive and robust microbial cell factories.

Growing evidence indicates that programmable post translational modification systems may support synthetic extracellular matrix engineering, biomolecular compartmentalization, and adaptive protein scaffolding within microbial environments (Fatima and Khare, 2018; Zhou et al., 2025; Deweid et al., 2019). Such systems could potentially improve metabolic efficiency by stabilizing biosynthetic complexes, reducing diffusion limitations, and enabling spatial substrate channeling. These emerging applications position MTGase at the interface of enzymology, systems biology, biomaterials engineering, and synthetic biotechnology.

### 4.3 Synthetic genetic circuits and dynamic regulation

The integration of MTGase with synthetic genetic circuits and dynamic regulatory systems may expand the programmability of microbial cell factories (Zhang X. et al., 2023; Yang T. et al., 2024). Synthetic biology has widely adopted CRISPR/dCas mediated transcriptional regulation, inducible promoter systems, quorum sensing circuits, and feedback/feedforward regulatory loops to optimize metabolic pathways and reduce cellular burden associated with heterologous expression. However, many transcription centered regulatory systems remain limited by delayed response kinetics, pathway instability, and insufficient adaptive control under industrial conditions.

At present, these regulatory technologies should be considered enabling strategies for MTGase control rather than established integrated MTGase circuits. CRISPR/dCas platforms enable programmable transcriptional activation or repression of target genes through guide RNA directed DNA binding. Integration of such systems with MTGase based post translational engineering may facilitate multi layered regulation combining transcriptional control with adaptive protein remodeling. Similarly, synthetic promoters and inducible expression systems may enable dynamic regulation of MTGase activity in response to metabolic or environmental signals.

Quorum sensing circuits and feedback regulation systems are also widely utilized for adaptive metabolic balancing and burden minimization (Ream and Prather, 2024). These systems may coordinate pathway activation according to cellular density or metabolic demand, thereby reducing unnecessary energy expenditure and improving production stability (Dinh and Prather, 2019). MTGase mediated structural stabilization and protein assembly may further enhance pathway robustness and metabolic organization within such dynamically regulated systems.

The convergence of synthetic genetic circuits with MTGase mediated post translational engineering may therefore support the development of programmable chassis systems capable of integrating metabolic sensing, adaptive expression control, and spatial catalytic organization (Casteleijn et al., 2025). Such strategies are particularly relevant for precision fermentation, industrial biotechnology, and therapeutic microbial engineering applications requiring robust and dynamically responsive biological systems.

### 4.4 Metabolic engineering and spatial biocatalysis

Spatial organization and compartmentalization of metabolic pathways represent important emerging strategies in synthetic biology aimed at improving biosynthetic efficiency and reducing pathway interference (Volk et al., 2022; Yang et al., 2026). Traditional metabolic engineering approaches often rely on overexpression of pathway enzymes without sufficient control over spatial localization or substrate accessibility, resulting in metabolic burden, intermediate diffusion losses, and reduced catalytic efficiency (Yang et al., 2026; Chai et al., 2025).

MTGase mediated protein assembly systems may support spatial biocatalysis through programmable enzyme clustering, scaffold formation, and biomolecular compartmentalization (Fatima et al., 2021a; Mondal et al., 2023; Sun et al., 2025). However, direct demonstration of MTGase mediated intracellular enzyme clustering or substrate channeling in microbial cell factories remains limited. By enabling covalent stabilization of biosynthetic complexes, MTGase may facilitate substrate channeling and improve metabolic flux efficiency within engineered microbial systems. Such strategies may reduce accumulation of toxic intermediates, minimize competing pathway interactions, and improve catalytic productivity.

Synthetic organelle like systems and orthogonal metabolic architectures are increasingly being explored for adaptive microbial engineering and precision fermentation applications (Rasor et al., 2025). MTGase assisted structural organization may contribute to the development of artificial catalytic compartments capable of selectively concentrating enzymes and metabolites within defined spatial regions. These approaches may mimic natural cellular compartmentalization while providing programmable control over metabolic organization (Liu et al., 2023; Deng et al., 2025).

Furthermore, spatially organized biocatalytic systems may improve industrial scalability and process stability by enabling enhanced catalytic durability, pathway insulation, and environmental responsiveness. The integration of MTGase with metabolic engineering frameworks therefore represents a promising strategy for developing adaptive microbial biofactories with improved efficiency and robustness.

### 4.5 Nanobiomaterials and adaptive catalytic systems

Integration of MTGase with nanostructured carriers and biomaterial matrices has been experimentally investigated as a strategy to improve enzyme immobilization, catalytic retention, structural organization, and material crosslinking. Independent studies provide quantitative evidence for these applications. Ma et al. (2019) expressed *S. mobaraensis* MTGase in *Lactococcus lactis* and subsequently immobilized the purified enzyme on porous magnetic Fe3O4 nanoparticles. Immobilization increased the measured activity from 27.6 ± 0.5 U/mg to 29.1 ± 0.4 U/mg, while the immobilized enzyme retained 67.2% of its initial activity after ten reuse cycles, demonstrating improved catalyst recovery and repeated use, although progressive activity loss remained evident (Ma et al., 2019). Gajšek et al. (2019) similarly investigated transglutaminase immobilization on surface modified magnetic nanoparticles and reported high immobilization efficiency, enhanced apparent activity, and improved thermal and operational stability, further supporting magnetic carriers as a practical strategy for enzyme reuse (Gajšek et al., 2019).

MTGase mediated conjugation has also been applied to site specific immobilization of other enzymes. (Wang et al., 2019) used MTGase to covalently immobilize an engineered enterokinase on amine functionalized magnetic nanoparticles. Approximately 90% of the original enzymatic activity was retained after immobilization, and more than 85% remained after prolonged storage or repeated use over 1 month (Wang et al., 2019). More recently, MTGase mediated site-specific immobilization of glutamine tagged PNGase F on magnetic particles preserved enzymatic performance while improving reusability and thermal stability (Zhang et al., 2023c). These studies demonstrate that MTGase can serve not only as the immobilized enzyme itself but also as a catalyst for controlled assembly of other biocatalysts.

Independent biomaterial studies further illustrate the structural capabilities of MTGase. Trengove et al. (2021) combined MTGase with gelatin methacryloyl hydrogels and demonstrated that enzymatic crosslinking was compatible with photocrosslinking and increased adhesion to human cartilage *ex vivo* from 2.87 ± 0.9 kPa to 5.82 ± 1.4 kPa. The system also showed slower degradation and maintained metabolic activity of encapsulated human adipose derived stem cells, providing direct evidence for MTGase mediated reinforcement of a biologically compatible hydrogel system (Trengove et al., 2021). In another approach, an engineered MTGase reactive Link module was incorporated into a hyaluronan gelatin hydrogel, enabling covalent network formation while retaining hyaluronan binding and demonstrating biocompatibility and degradability in cell and animal studies (Okawa et al., 2022).

Studies by Fatima et al. extended MTGase containing nanomaterial systems toward hydrogel scaffold formation and biomedical applications (Fatima et al., 2020; Fatima et al., 2021a). MTGase immobilization on multiwalled carbon nanotubes was evaluated for crosslinked hydrogel fabrication (Fatima et al., 2020), whereas MTGase nanoflowers were subsequently investigated in breast cancer related theranostic models (Fatima et al., 2021a). Transglutaminase polyethyleneimine nanoflowers were further examined as carriers for anti miR 210 delivery in glioblastoma cells (Mondal et al., 2023). These studies demonstrate distinct capabilities including enzyme immobilization, material assembly, nanoscale organization, and molecular delivery. However, they represent application specific proof of concept systems and should not be interpreted as direct validation of intracellular MTGase control in microbial cell factories.

Several technical limitations remain across these platforms. Immobilization performance depends on carrier chemistry, enzyme orientation, loading density, substrate accessibility, diffusion, and preparation reproducibility. High immobilization efficiency does not necessarily translate into higher process productivity, and repeated reuse can result in progressive activity loss. Nanostructured systems additionally require control of particle size, morphology, enzyme loading, batch variation, and biological compatibility. Importantly, results obtained from extracellular biomaterials or mammalian cell systems cannot be directly extrapolated to microbial intracellular environments, where proteostasis, substrate competition, enzyme localization, and host fitness impose additional constraints. Therefore, current evidence supports MTGase integration with nanobiomaterials primarily for enzyme immobilization, molecular assembly, and material crosslinking, whereas its use for intracellular spatial organization or dynamic metabolic control remains to be experimentally established.

### 4.6 AI driven enzyme and pathway optimization

The rapid maturation of computational and data driven enzyme engineering is shifting protein optimization from extensive empirical screening toward more focused prediction and experimental validation (Gong et al., 2024; Volk et al., 2026). Data driven approaches can integrate sequence, structural, biochemical, and mutational information to identify function enhancing variants and reduce the experimental search space (Singh N. et al., 2025; Docter et al., 2026). (Jiang et al., 2023) highlighted how machine learning and related data driven methods can be used to connect protein sequence and structure with catalytic properties, while also emphasizing limitations imposed by sparse and heterogeneous enzyme datasets (Jiang et al., 2023). Wang et al. (2023) further examined the potential of *de novo* protein design for industrial and food enzymes, noting that broader sequence exploration could generate functions inaccessible through conventional point mutagenesis, although limited training data and incomplete relationships between enzyme substrate binding and catalytic activity remain major constraints (Wang et al., 2023).

Recent experimental developments illustrate the movement from conceptual frameworks toward quantitative enzyme optimization. Zheng et al. (2025) introduced the iCASE strategy, which combines structure based descriptors with supervised machine learning to predict enzyme function and fitness across enzymes of different structural classes. The approach achieved simultaneous improvements in stability and activity and was experimentally extended to MTGase, laccase, and PET hydrolase, supporting its broader applicability to industrial enzyme engineering (Zheng et al., 2025). More recent approaches such as Hybridized Evolution Accelerating Thermostability (HEAT) further combine evolutionary search with multiobjective optimization to explore thermostability related sequence space, reflecting a broader trend toward computational strategies that balance multiple enzyme properties rather than optimizing a single parameter (Browning et al., 2026).

Generative and AI assisted molecular design are also expanding beyond individual enzyme mutations. *De novo* design approaches can explore protein sequence and structural space beyond naturally occurring variants, whereas AI driven peptide and conjugate design has been applied to prediction of molecular interactions, linker optimization, and *in silico* screening of functional scaffolds. Pandey et al. (2026), for example, discussed the growing role of AI and deep learning in the design of nucleotide peptide and peptide drug conjugates, illustrating how computational prediction can guide molecular recognition and conjugation strategies relevant to protein engineering and bioconjugation. These developments provide useful design principles for MTGase mediated conjugation systems, although direct experimental transfer to MTGase remains to be established (Pandey et al., 2026).

For microbial cell factories, computational optimization extends beyond the enzyme itself. Machine learning, metabolic modeling, and pathway prediction can be used to prioritize pathway interventions, estimate flux redistribution, and guide iterative Design Build Test Learn cycles (Xie and Tummala, 2025; Liao et al., 2025; Gao et al., 2025). However, computational prediction should be interpreted as a means of prioritizing experimentally testable interventions rather than as evidence of improved biological performance. For MTGase specifically, the strongest evidence currently supports structure guided mutagenesis, molecular docking, molecular dynamics, and machine learning assisted optimization of thermostability and substrate preference. Generative design, autonomous pathway control, and digital twin integration remain prospective areas that require direct experimental validation in MTGase based microbial systems.

### 4.7 Integrated microbial cell factory framework

The construction of high performance microbial cell factories now relies on hierarchical engineering strategies that operate across multiple biological scales, from genetic parts and metabolic pathways to chromosome level and genome wide organization. In the case of MTGase production, improvements in promoter strength, signal peptide performance, secretion pathways, codon usage, and gene copy number have significantly increased industrial output in production hosts such as *S. mobaraensis* and heterologous systems (Zhou et al., 2025; Chang et al., 2025; Aqeel et al., 2024; Fatima et al., 2021b). To further enhance efficiency and reduce metabolic burden, researchers are also developing synthetic microbial consortia in which different strains distribute pathway tasks across a cooperative community. This division of labor can improve productivity, facilitate the co production of difficult compounds, and increase robustness under industrial conditions. In parallel, biofoundries are providing high throughput and automated platforms for strain construction, screening, and optimization, helping overcome traditional bottlenecks in scale up and implementation.

The development of advanced microbial cell factories has progressed from relatively simple genetic modifications to sophisticated systems metabolic engineering informed by synthetic biology and artificial intelligence (Zheng et al., 2024). By exploiting enzymes such as asparaginase, amidase, and microbial transglutaminase, researchers are building adaptive biotechnological platforms for industrial catalysis, pharmaceutical manufacturing, and the generation of programmable biomaterials (Figure 2). Figure 2 presents an integrated framework for engineering adaptive microbial cell factories by connecting multiomics, AI, synthetic biology, and amide transforming enzymes within a coordinated workflow. The framework begins with environmental inputs and system sensing (Stage 1), followed by multiomics data acquisition (Stage 2) through genomics, transcriptomics, proteomics, metabolomics, and fluxomics to define regulatory networks, enzyme activity, metabolic flux, and cellular state. These data inform AI assisted computational design (Stage 3), including machine learning, predictive modeling, structure prediction, and pathway optimization, which can guide interventions implemented through the synthetic biology toolkit (Stage 4). A central component is the amide transforming enzyme reconfiguration hub (Stage 5), where enzyme activity, localization, specificity, or organization may be engineered to influence protein modification, crosslinking, immobilization, metabolic channeling, and spatial catalytic organization. These interventions ultimately affect the microbial cell factory (Stage 6) and its bioproduction outputs (Stage 7), including proteins, fine chemicals, biofuels, biomaterials, and other value-added products.

**FIGURE 2:**
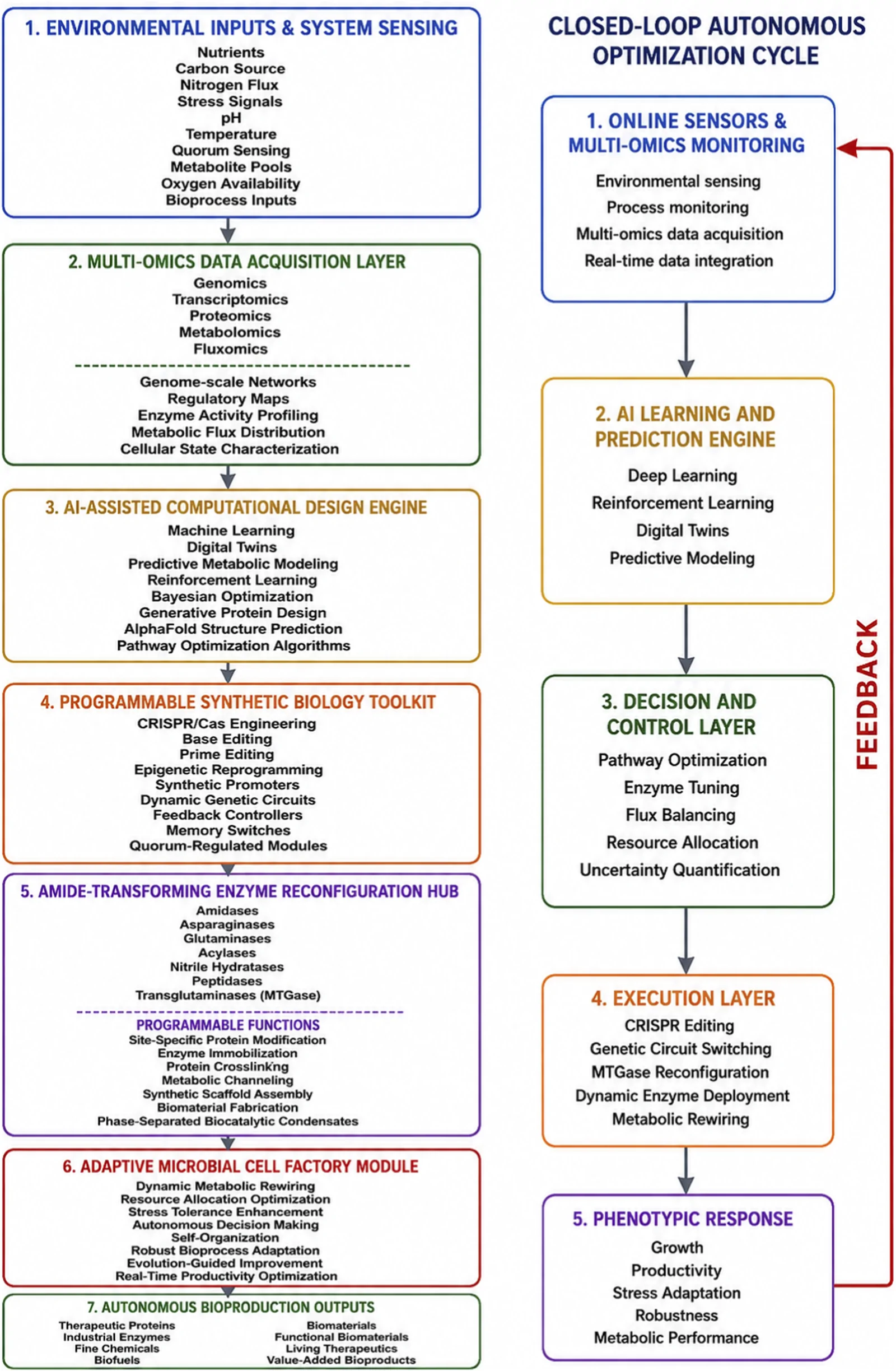
Integrated framework for AI guided engineering of adaptive microbial cell factories through multi omics and programmable amide transforming enzymes. Schematic overview of a systems level engineering framework integrating environmental sensing, multi omics profiling, AI assisted computational design, programmable synthetic biology, and amide transforming enzyme reconfiguration to enable adaptive microbial cell factories. The framework illustrates the progression from environmental inputs through data driven design, dynamic genetic and enzymatic reprogramming, autonomous cellular adaptation, and scalable bioproduction. The closed loop optimization cycle combines real time sensing, predictive learning, decision making, and iterative execution to continuously optimize microbial performance, robustness, and productivity.

The framework further extends this sequence into a closed loop optimization cycle in which online sensing and multiomics monitoring inform AI based prediction, pathway and enzyme level decisions, and subsequent genetic or enzymatic interventions. The resulting phenotypic response, including growth, productivity, stress adaptation, robustness, and metabolic performance, is then fed back into the sensing system for iterative refinement. Conceptually, the framework differs from conventional AI assisted strain engineering by positioning amide transforming enzymes as a potential interface between computational prediction and protein level metabolic control. Enzyme abundance, localization, substrate specificity, catalytic activity, and assembly are therefore considered additional control variables. Importantly, dynamic intracellular MTGase regulation and autonomous feedback control remain prospective and require experimental validation, making the framework both a conceptual model and a roadmap for future engineering studies.

Modern microbial cell factory design draws heavily on systems biology, metabolic control analysis, and synthetic regulation to optimize flux distribution, reduce by product formation, and enhance host robustness (Xu et al., 2024). Engineering strategies now extend beyond simple overexpression of individual genes and instead involve coordinated regulation of transport systems, secretion pathways, redox balance, precursor supply, and stress tolerance (Cao et al., 2026). For enzymes such as asparaginase and MTGase, successful production depends not only on the intrinsic quality of the enzyme but also on the physiological compatibility of the host (Fatima and Khare, 2018; Jana et al., 2025; Chand et al., 2020; Fisher and Wray Jr, 2002). For example, glutaminase free or low glutaminase L-asparaginase production in *Streptomyces* and related organisms has been improved through substrate selection, fermentation optimization, and low cost nutrient sourcing, including agro industrial substrates such as soybean meal and wheat bran.

The convergence of MTGase based platforms with CRISPR enabled regulation and AI guided protein engineering provides a strong conceptual and technological foundation for next-generation biomanufacturing, with several future directions standing out as especially important. Intelligent regulation will emerge through the integration of intracellular biosensors, dynamic feedback loops, and synthetic gene circuits, enabling autonomous metabolic control in response to both intracellular and environmental signals. In parallel, expanding the engineering toolbox to non model microorganisms will unlock hosts with distinctive metabolic capabilities, greater stress tolerance, and broader substrate utilization profiles than those typically found in conventional laboratory organisms. At the same time, sustainable bioprocessing will heavily rely on renewable feedstocks and agro industrial waste streams, including starch rich and protein rich residues such as potato waste, to improve process economics while reducing environmental impact.

### 4.8 Comparative roles and engineering requirements of amide transforming enzymes

Amidases, asparaginases, glutaminases, and MTGase contribute distinct but complementary functions to microbial biotechnology and cell factory engineering. Amidases support pollutant degradation, fine chemical synthesis, and engineered biodegradation pathways, while asparaginases contribute to nitrogen metabolism, food processing, and therapeutic biotechnology (Voor et al., 2025; Rotilio et al., 2025; Marti et al., 2024; Jana et al., 2025; Alam et al., 2018; Abdallah and Bello, 2026). Glutaminases primarily regulate glutamine utilization, carbon nitrogen balance, and metabolic flux distribution (Gibhardt et al., 2026). These enzymes therefore function predominantly as catalytic or metabolic modules within engineered microbial systems.

Computational and protein engineering approaches are increasingly being applied to improve the performance of these enzymes. For amidases, data driven methods can assist in predicting substrate specificity, stereoselectivity, and structural determinants associated with substrate access and catalytic behavior. Asparaginase engineering similarly employs rational design, molecular modeling, and computationally assisted mutagenesis to improve catalytic activity, stability, and substrate selectivity while reducing undesirable glutaminase activity (Jana et al., 2025; Alam et al., 2018; Avramis and Tiwari, 2006). Such approaches can complement fermentation optimization and host engineering strategies aimed at improving enzyme production and process performance.

MTGase differs mechanistically from these hydrolytic enzymes because it catalyzes covalent protein crosslinking and therefore provides access to protein modification, molecular assembly, and structural organization (Fatima and Khare, 2018; Zhou et al., 2025). Recent computational studies have directly targeted MTGase properties including thermostability, activity, and substrate preference. Machine learning guided approaches have identified variants such as S199E with improved thermal stability and catalytic performance (Song et al., 2026), while structure informed computational engineering has been used to modify substrate recognition and prioritize function enhancing substitutions (Wang et al., 2025). These studies demonstrate experimentally supported applications of computational engineering to MTGase, although broader control of intracellular crosslinking remains unresolved.

From a synthetic biology perspective, the principal distinction among these enzyme classes therefore lies in the level at which they operate. Amidases, asparaginases, and glutaminases primarily influence substrate conversion, nutrient utilization, and metabolic state, whereas MTGase acts directly on protein organization through covalent post translational modification. Future microbial cell factory designs may therefore benefit from combining hydrolytic enzymes for metabolic sensing and pathway regulation with more selective protein level engineering strategies. However, such hierarchical integration will require experimental control of substrate specificity, enzyme activity, localization, host burden, and interaction with endogenous proteins before integrated MTGase based systems can be considered established (Fatima and Khare, 2018; Song et al., 2026).

### 4.9 Digital twin biofactories and autonomous design build test learn platforms

The future of microbial cell factory engineering is moving toward autonomous and self-optimizing biological manufacturing systems. A particularly transformative concept is the development of digital twin biofactories, in which computational models continuously simulate and predict the behavior of biological systems in real time.

Digital twins combine multi omics datasets, metabolic network models, machine learning algorithms, bioprocess sensors, and predictive analytics to generate virtual representations of microbial production systems (Shukla et al., 2026). These computational frameworks allow researchers to evaluate pathway performance, predict metabolic bottlenecks, estimate product yields, and identify optimal engineering strategies before conducting physical experiments.

Integration of digital twin technologies with MTGase enabled synthetic biology platforms may provide unprecedented opportunities for adaptive process optimization. Real time monitoring of metabolic activity, protein assembly dynamics, and environmental conditions could enable autonomous adjustment of gene expression, enzyme activity, and pathway organization. Such systems may facilitate continuous learning and optimization through iterative Design Build Test Learn (DBTL) cycles (Cochrane et al., 2026; Douchi et al., 2026).

The convergence of digital twins, robotic biofoundries, automated strain engineering, and AI guided decision making represents a major step toward self-driving laboratories and intelligent microbial manufacturing systems. These technologies have the potential to significantly reduce development costs, shorten engineering timelines, and accelerate commercialization of sustainable bioprocesses.

## 5 Discussion

The central potential presented by MTGase is its ability to extend microbial engineering from gene and pathway level regulation to direct covalent control of protein organization. However, this capability also introduces challenges that are less prominent for conventional metabolic enzymes. Broad substrate accessibility can generate unintended crosslinks, irreversible bond formation limits reversibility, and intracellular deployment requires substantially tighter spatial and temporal control than conventional extracellular applications. Consequently, the value of MTGase in microbial cell factories will depend less on maximizing catalytic activity than on achieving selective, stoichiometrically controlled, and condition dependent protein modification (Beitello et al., 2026; Kieliszek, 2026; Fatima and Khare, 2018).

Synthetic genetic circuits, CRISPR mediated regulation, inducible promoter systems, and feedback mechanisms provide potential upstream approaches for controlling MTGase expression and deployment (Sarkar et al., 2023; Wu et al., 2020). However, transcriptional control alone does not address broad substrate recognition, uncertain intracellular crosslink stoichiometry, or possible disruption of endogenous proteostasis. Strategies such as orthogonal recognition tags, substrate specificity engineering, proenzyme based activation, spatial restriction, and quantitative control of enzyme to substrate ratios may therefore be required to establish reliable intracellular use (Akbari et al., 2021; Song et al., 2026; Deweid et al., 2019).

Experimental studies further show that MTGase can retain catalytic function when incorporated into immobilization matrices, nanostructured carriers, and biomaterial systems (Fatima et al., 2020; Fatima et al., 2021a). These findings support applications in enzyme stabilization, material crosslinking, and spatial organization of catalytic systems. However, evidence from biomaterial and mammalian cell models should not be directly extrapolated to intracellular microbial engineering. Reproducibility can be influenced by enzyme loading, carrier chemistry, substrate accessibility, diffusion constraints, particle or scaffold heterogeneity, and retention of catalytic activity. Industrial translation also remains uncertain because performance under large scale fermentation conditions has not been comprehensively established. Thus, nanobiomaterial integration provides experimental support for immobilization and structural organization, whereas its role in intracellular metabolic control remains largely prospective.

Computational approaches may assist in addressing some of these limitations, but their functions should be distinguished clearly. AlphaFold based structure prediction provides structural models that can support identification of candidate mutation sites, whereas molecular docking and molecular dynamics can examine substrate interactions and conformational behavior. Machine learning approaches can further prioritize mutations associated with properties such as stability or substrate preference (Zheng et al., 2025). Importantly, computational prediction does not demonstrate improved catalytic performance. Experimentally validated changes in thermal stability, activity, or substrate specificity therefore remain essential for assessing the value of these approaches. Generative protein design and digital twin strategies represent emerging opportunities, but their direct validation for MTGase engineering remains limited.

Several barriers continue to restrict broader implementation of MTGase in microbial systems (Gilbert and Ellis, 2018). These include metabolic and proteostatic burden, unintended host interactions, genetic instability, limited temporal control of catalytic activity, chassis dependent behavior, and uncertain performance during industrial scale up (Kim et al., 2025; Ren et al., 2025; Liu et al., 2025). Future studies should therefore evaluate MTGase using quantitative criteria such as off target crosslinking, crosslink occupancy, host fitness, catalytic retention, expression stability, and productivity under controlled fermentation conditions. Orthogonal substrate systems, burden responsive regulation, spatial confinement, and computationally guided variant selection may help address these limitations (Guo et al., 2026; Qin et al., 2026; Cho et al., 2022). Together, these approaches therefore represent interconnected levels of an engineering hierarchy spanning genetic regulation, protein level control, spatial organization, and computational optimization rather than independent technological themes.

Taken together, current evidence supports MTGase as a versatile crosslinking enzyme with demonstrated utility in protein modification, immobilization, biomaterial formation, and selected engineered systems (Zhou et al., 2025). Its broader role in microbial synthetic biology will depend on whether these capabilities can be translated into selective and controllable intracellular function. Progress in this area should therefore be judged by experimental demonstration of specificity, temporal regulation, stoichiometric control, host compatibility, reproducibility, and process robustness rather than by the breadth of potential applications proposed.

## 6 Current limitations, engineering bottlenecks, and mitigation strategies

Despite the potential of MTGase in engineered microbial systems, intracellular implementation presents challenges that differ substantially from conventional extracellular or process applications. Major barriers include unintended crosslinking of host proteins, variable crosslink stoichiometry, metabolic and proteostatic burden, insufficient temporal control of MTGase activity, chassis dependent behavior, and loss of performance during scale up. Thus, development of MTGase as an intracellular synthetic biology tool requires not only improved catalytic activity, but also substrate selectivity, tunability, spatial restriction, and process compatible control.

A major limitation arises from the broad substrate accessibility of MTGase. Because the enzyme catalyzes covalent bond formation between accessible glutamine and lysine residues, unrestricted intracellular activity may modify unintended host proteins, generate aberrant protein assemblies, interfere with protein turnover, and disrupt proteostasis. This issue becomes particularly important when MTGase is considered for intracellular structural or metabolic organization, where endogenous proteins create a substantially more complex substrate environment than purified industrial substrates. The biological consequence should therefore be considered not simply as off target crosslinking, but as a potential disturbance of cellular proteostasis and fitness.

Several engineering strategies may reduce this risk. Orthogonal substrate recognition systems could restrict MTGase activity to engineered peptide tags or defined glutamine and lysine containing motifs introduced into target proteins. Selective protein conjugation using engineered recognition sequences has already demonstrated that MTGase substrate preference can be redirected toward defined molecular targets. Structure guided mutagenesis, directed evolution, and high throughput screening may further reduce substrate promiscuity. Spatial restriction through secretion, membrane anchoring, synthetic scaffolds, encapsulation, or compartmentalization could also separate MTGase activity from the bulk intracellular proteome. Expression of MTGase as an inactive precursor provides an additional safeguard. *Streptomyces mobaraensis*, for example, naturally produces MTGase as a proenzyme in which the N terminal proregion suppresses premature catalytic activity while supporting folding and secretion.

A second barrier is the difficulty of controlling crosslink stoichiometry within a complex microbial chassis. Crosslinking depends on enzyme abundance, accessibility of reactive residues, local protein concentration, cellular localization, substrate competition, and physicochemical conditions. Increasing MTGase expression therefore does not necessarily produce a proportional increase in the desired product. Instead, mixtures of unmodified, singly modified, multiply crosslinked, or polymerized species may arise. This becomes particularly problematic for metabolic scaffolding or intracellular organization, where uncontrolled network formation could alter enzyme mobility or generate nonoptimal assemblies.

Mitigation requires control at both substrate and expression levels. Defined recognition tags, engineered linker geometry, controlled target protein ratios, and substrate selective MTGase variants could constrain the number and position of crosslinks. Quantitative proteomics and crosslinking mass spectrometry could be incorporated into Design Build Test Learn workflows to measure site occupancy and identify unintended crosslinks. Promoter strength, ribosome binding site activity, gene copy number, and degradation tags could likewise be tuned to maintain MTGase within an appropriate operating range. The objective should therefore be quantitative control of enzyme to substrate ratios and crosslink occupancy rather than simple maximization of MTGase expression.

A further unresolved challenge is temporal control of catalytic activity. Inducible promoters can regulate MTGase expression, but transcriptional control does not immediately regulate enzyme molecules that have already accumulated. This distinction is important because MTGase mediated covalent crosslinks are effectively irreversible under normal cellular conditions. CRISPR/dCas systems, inducible promoters, quorum sensing circuits, and feedback controllers are established tools for regulating heterologous gene expression, but their direct integration with MTGase remains largely prospective.

Effective temporal regulation may therefore require control at both transcriptional and protein levels. Tightly regulated promoters could delay MTGase expression until sufficient biomass has accumulated, separating growth from the production phase and reducing metabolic burden. At the protein level, proenzyme architectures, removable inhibitory domains, degradation tags, or localization switches could provide faster control overactive enzyme concentration. The native MTGase propeptide is particularly relevant because it demonstrates that catalytic activity can be suppressed during biosynthesis and activated subsequently. Future systems could couple this principle to fermentation compatible signals such as nutrient status, cell density, metabolic state, or process variables. However, a robust intracellular MTGase activity switch validated across industrial fermentation scales has not yet been established.

Metabolic and proteostatic burden represents another important constraint. Simultaneous expression of MTGase, engineered substrates, sensing modules, CRISPR regulators, and feedback circuitry imposes demands on transcriptional, translational, energetic, and protein folding resources. Potential mitigation strategies include low copy genomic integration instead of high copy plasmids, delayed MTGase expression after biomass accumulation, burden responsive regulation, tunable degradation, secretion when intracellular activity is unnecessary, and division of functions across microbial consortia.

MTGase performance is also likely to vary among *Streptomyces*, *E. coli*, *Bacillus*, yeast, and other microbial hosts because secretion capacity, protease activity, protein folding machinery, intracellular redox conditions, cell envelope organization, and stress responses differ among chassis. Heterologous production has been demonstrated in several hosts, but successful enzyme production does not itself establish reliable intracellular crosslinking. During scale up, additional variation arises from oxygen transfer limitations, nutrient gradients, pH fluctuations, shear, and population heterogeneity.

Potential strategies include chromosomal integration to improve genetic stability, host specific promoter and secretion optimization, regulatory systems that remain functional under heterogeneous reactor conditions, online monitoring of biomass and metabolic state, and scale down bioreactor models that reproduce industrial oxygen and nutrient gradients before pilot scale testing. Digital twin and model predictive control approaches may eventually support this optimization, but their integration with MTGase based systems remains emerging rather than established.

Highly engineered microorganisms carrying protein remodeling systems also raise biosafety concerns related to genetic stability, containment, environmental persistence, and long term predictability. Appropriate safeguards may include auxotrophic containment, dependence on nonnatural metabolites, kill switches, genome integrated rather than mobile genetic constructs, and physical containment at the bioprocess level. For therapeutic or environmental applications, these safeguards would require long term validation because mutations that reduce the fitness cost of containment systems may compromise their reliability.

Taken together, the principal challenge for MTGase based synthetic biology is not simply increasing enzyme activity, but achieving selective, stoichiometrically controlled, temporally regulated, and industrially robust crosslinking. Engineered recognition tags, protein engineering, proenzyme-based suppression, inducible expression, regulatory circuits, compartmentalization, and quantitative systems biology each address part of this challenge, but no single platform currently integrates all of these properties in an industrially validated microbial chassis. This gap defines a key research priority for advancing MTGase from a broadly acting crosslinking enzyme toward a controllable post translational engineering tool.

## 7 Knowledge gaps, outstanding questions, and future perspectives in programmable biocatalytic systems

Despite significant advances in synthetic biology, metabolic engineering, artificial intelligence guided protein design, and programmable biocatalysis, several critical knowledge gaps continue to limit the development and large-scale implementation of adaptive microbial cell factories (Ezeako et al., 2025). While MTGase has emerged as a promising programmable molecular platform for post translational engineering and biomolecular assembly, many fundamental biological, engineering, and translational questions remain unresolved.

One major challenge concerns the integration of programmable protein level regulation with existing transcriptional and translational control systems. Current synthetic biology platforms predominantly rely on gene expression-based regulation through synthetic promoters, CRISPR mediated control, and transcriptional feedback circuits (Shan et al., 2026). Although MTGase enables direct protein remodeling and covalent network formation, the long term consequences of incorporating programmable post translational engineering into complex microbial systems remain poorly understood. Future studies are needed to determine how protein level engineering interfaces with cellular signaling networks, metabolic homeostasis, proteostasis mechanisms, and host fitness under industrial fermentation conditions.

Another important knowledge gap involves the development of orthogonal MTGase based protein engineering systems. While MTGase exhibits broad substrate compatibility, achieving highly selective and programmable protein modifications within complex intracellular environments remains challenging. The design of orthogonal substrate recognition modules engineered peptide tags, and programmable crosslinking architecture could significantly improve the precision and predictability of MTGase mediated synthetic biological systems (Nagamune, 2017). Such advances may facilitate the construction of sophisticated protein circuits, dynamic biomolecular assemblies, and programmable catalytic interfaces with minimal interference from endogenous cellular processes.

The scalability of MTGase enabled spatial biocatalysis also remains largely unexplored. Although enzyme clustering, biomolecular compartmentalization, and synthetic metabolic scaffolding have demonstrated promising proof of concept results, their performance under industrial scale bioprocessing conditions is still uncertain (Cao et al., 2025). Additional research is required to determine whether MTGase mediated spatial organizations can consistently improve metabolic flux, reduce pathway interference, enhance substrate channeling, and maintain long term stability during large scale fermentation and continuous biomanufacturing operations.

Artificial intelligence is expected to play a key role in addressing these limitations. However, current AI driven protein engineering platforms remain largely focused on structural prediction and enzyme optimization (Chen et al., 2025). Future computational frameworks must move beyond individual enzyme design toward integrated modeling of dynamic cellular behavior, metabolic networks, protein interaction systems, and adaptive regulatory circuits. The development of digital twin microbial systems capable of simulating real time cellular responses may provide new opportunities for predictive engineering and autonomous optimization of microbial biofactories (Lv et al., 2025; Khan and Khan, 2025).

Finally, important translational questions remain regarding biosafety, regulatory compliance, genetic stability, and environmental containment of highly engineered microbial systems. As synthetic biology progresses toward increasingly autonomous and adaptive biological platforms, robust biosafety mechanisms, programmable containment systems, and standardized regulatory frameworks will become essential for commercial deployment. The convergence of AI and synthetic biology lowers technical barriers to biological design and manipulation, which raises legitimate dual use concerns (Groff Vindman et al., 2025). The same tools that support beneficial advances in medicine, agriculture, and manufacturing could also be misused to design harmful biological systems or facilitate the synthesis of dangerous agents. Because the risk resides not only in physical materials but also in models, datasets, and distributed automated workflows, biosecurity strategies must evolve accordingly. Measures such as secure data governance, DNA synthesis screening, access controls, and responsible publication practices will be essential. Addressing these challenges will be critical for translating programmable biocatalytic systems from laboratory scale demonstrations into sustainable industrial biotechnology applications (Cochrane et al., 2026).

## 8 Conclusion

Amide transforming enzymes occupy complementary roles in microbial biotechnology, ranging from nitrogen and xenobiotic metabolism to selective chemical conversion. MTGase is distinguished by its acyl transfer activity, which enables covalent protein crosslinking, protein stabilization, molecular assembly, enzyme immobilization, and spatial organization of catalytic systems. Advances in synthetic biology and microbial engineering have broadened the context in which MTGase can be investigated, including integration with synthetic genetic circuits, CRISPR mediated regulation, biomaterial assisted catalysis, spatial biocatalysis, and AI guided protein engineering (Zhou et al., 2025; Sarkar et al., 2023; Wu et al., 2020; Jun et al., 2025). These approaches could extend MTGase applications beyond conventional industrial crosslinking toward more precise control of protein organization and metabolic function.

However, intracellular application of MTGase remains constrained by substrate promiscuity, off target protein modification, uncertain crosslink stoichiometry, irreversible catalytic effects, metabolic and proteostatic burden, limited temporal control, chassis dependence, and industrial scale up. Progress will therefore depend on experimentally establishing orthogonal substrate recognition, temporally controlled activation, spatial restriction, quantitative monitoring of crosslink occupancy, and robust performance under industrial fermentation conditions. Digital twin modeling, robotic biofoundries, and iterative Design Build Test Learn workflows may assist the systematic evaluation of enzyme variants, regulatory strategies, and process conditions (Kim et al., 2025; Xu et al., 2024; Cao et al., 2026; Mariam et al., 2025), while integration with spatially organized catalytic systems and AI guided optimization may further support controlled protein level engineering (Khan and Khan, 2025). Such advances could support applications in therapeutics, biomaterials, specialty chemicals, renewable fuels, and engineered living materials (Chen et al., 2025). Ultimately, the broader value of MTGase in synthetic biology will depend on demonstrating selective and controllable activity, reproducible performance, host compatibility, and scalability under industrially relevant conditions.
